# Efficacy of Chlorantraniliprole in Controlling Structural Infestations of the Eastern Subterranean Termite in the USA

**DOI:** 10.3390/insects8030092

**Published:** 2017-08-31

**Authors:** Susan C. Jones, Edward L. Vargo, T. Chris Keefer, Paul Labadie, Clay W. Scherer, Nicola T. Gallagher, Roger E. Gold

**Affiliations:** 1 Department of Entomology, The Ohio State University, Columbus, OH 43210, USA; 2 Department of Entomology, Texas A&M University, College Station, TX 77843, USA; ed.vargo@tamu.edu (E.L.V.); r-gold@tamu.edu (R.E.G.); 3 Department of Entomology, North Carolina State University, Raleigh, NC 27695, USA; paul_labadie@ncsu.edu; 4 Syngenta Crop Protection, LLC, Greensboro, NC 27409, USA; chris.keefer@syngenta.com (T.C.K.); nicky.gallagher@syngenta.com (N.T.G.); 5 Syngenta AG, 4002 Basel, Switzerland; clay.scherer@syngenta.com

**Keywords:** anthranilic diamide, microsatellite, *Reticulitermes flavipes*, Rhinotermitidae, termite control, termiticide

## Abstract

Subterranean termites are the most economically important structural pests in the USA, and the eastern subterranean termite, *Reticulitermes flavipes* (Kollar) (Dictyoptera: Rhinotermitidae) is the most widely distributed species. Soil treatment with a liquid termiticide is a widely used method for controlling subterranean termites in structures. We assessed the efficacy of a nonrepellent termiticide, Altriset^®^ (active ingredient: chlorantraniliprole), in controlling structural infestations of *R. flavipes* in Texas, North Carolina, and Ohio and determined the post-treatment fate of termite colonies in and around the structures. In all three states, microsatellite markers indicated that only one *R. flavipes* colony was infesting each structure. A single chlorantraniliprole treatment provided effective structural protection as there was no further evidence of termite activity in and on the majority of structures from approximately 1 month to 2 years post-treatment when the study concluded. Additionally, the treatment appeared to either severely reduce the infesting colony’s footprint at monitors in the landscape or eliminate colony members from these monitors. A supplemental spot-treatment was conducted at one house each in Texas and North Carolina at 5 and 6 months post-treatment, respectively; no termites were observed thereafter in these structures and associated landscaping. The number of colonies found exclusively in the landscape (not attacking the structure) varied among the states, with the largest number of colonies in Texas (0–4) and North Carolina (0–5) as compared to 0–1 in Ohio, the most northern state.

## 1. Introduction

Subterranean termites (Dictyoptera: Rhinotermitidae) are the most economically important structural pests in the USA, where wood is a dominant construction element. Native termites in the genus *Reticulitermes* occur in every state in the continental USA, and the eastern subterranean termite, *Reticulitermes flavipes* (Kollar), is the most widely distributed species. Its range encompasses the eastern half of the country [1] extending westward into numerous states as evidenced by numerous *R. flavipes* 16S haplotypes [2].

Soil treatment with a liquid termiticide is one of the primary methods for controlling subterranean termites. The termiticide is applied to the soil under and next to the building foundation to establish a chemical zone that is toxic to termites. Altriset^®^ with the active ingredient chlorantraniliprole was registered as a termiticide by the U.S. Environmental Protection Agency (EPA) in 2010, becoming the first liquid termiticide designated as a reduced risk insecticide [3]. It belongs to a new insecticide class, the anthranilic diamides [4,5], whose novel mode of action is activation of ryanodine receptors (large non-voltage-gated ion channels); they show high selectivity for ryanodine receptors of insects compared to mammals which leads to their low mammalian toxicity. Anthranilic diamides interfere with muscle contractions of target insects by stimulating the continual release of intracellular calcium, leading to calcium depletion, muscle paralysis, feeding cessation, lethargy, and ultimately death [4,5,6,7,8].

Laboratory studies have demonstrated that important pest termites are highly susceptible to chlorantraniliprole. These include several rhinotermitids, *R. flavipes* [9,10,11,12,13,14,15], *Coptotermes formosanus* Shiraki [11,16], and *Coptotermes gestroi* (Wasmann) [17,18], as well as a termitid, *Nasutitermes corniger* (Motschulsky) [19]. Chlorantraniliprole was quite toxic to *R. flavipes* with LD_50_s of 2.13 ng/worker at day 2 [9] and 0.98 ng/worker at day 7 [11]. Termites moved very slowly and sluggishly during the incipient phase of chlorantraniliprole intoxication [10]. Lethargic walking was followed by moribundity and death of the workers. Furthermore, *R. flavipes* ceased feeding soon after chlorantraniliprole exposure in laboratory bioassays [13], presumably due to paralysis of the workers’ large mandibular muscles [5]. Termites also reduced activities such as walking, trail following, grooming, and tunneling. Symptomatic individuals clustered together and remained inactive until their death 3–5 days later.

In a study that simulated commercial termiticide applications, chlorantraniliprole showed high persistence in four Midwestern soils for 705 days with the data best fitting a zero-order degradation kinetic model [12]. Treated soil sampled periodically from 0 to 705 days consistently caused high mortality of *R. flavipes*, and termites were unable to completely penetrate 50 mm columns of treated soil.

The objective of our study was to evaluate the efficacy of chlorantraniliprole in controlling structural infestations of *R. flavipes* and to determine the post-treatment fate of termite colonies in and around each structure. In order to accommodate common foundation types, which differ regionally in the USA, our study included structures in three states.

## 2. Materials and Methods

We evaluated a total of 12 free-standing residences that were infested with *R. flavipes*. These included four structures with representative foundation types in three regions of the country: monolithic slabs in College Station, TX; crawl spaces in Raleigh, NC; and basements in Columbus, OH. We prepared a diagram of each structure that depicted the linear dimensions and shape of the foundation. The main level of the structures averaged (±SD) 202 ± 88 m^2^ in TX, 140 ± 35 m^2^ in NC, and 133 ± 39 m^2^ in OH.

At study initiation (2012–2013), we conducted a comprehensive visual termite inspection augmented with a moisture meter for each structure, and we documented all interior and exterior locations with termite activity on the aforementioned house diagram. In order to collect termite samples (~20+ workers) from infestation points in and on the structure while minimizing wood damage, we installed auxiliary termite monitors inside structures in North Carolina and Ohio: wood stakes were inserted in the soil within crawl spaces, and plastic-wrapped corrugated cardboard pieces were affixed to wood members in basements. We inspected the structure and collected termite samples on a monthly basis for ~1–8 months pre-treatment. Termites from each collection point were placed into a vial filled with 100% ethanol and the sample was held for genetic analyses, which are described below.

**Figure 1:**
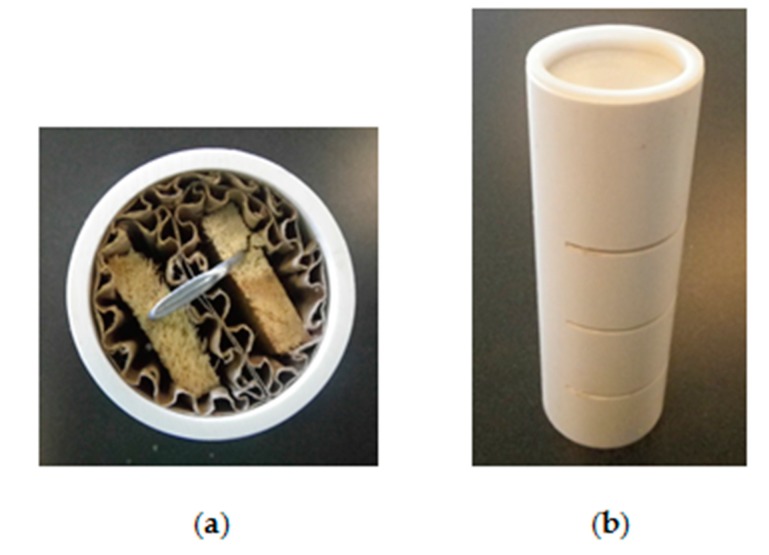
In-ground termite monitor (IGM): (**a**) Internal cellulose components consisting of two pine wood strips wrapped with corrugated cardboard; (**b**) PVC housing and cap.

In conjunction with the augmented structural inspection, we installed numerous in-ground termite monitors (IGMs) around each structure for the purpose of collecting termite samples for genetic analyses. Each IGM consisted of two 18-cm-long pine wood strips wrapped with corrugated cardboard and fitted inside a polyvinyl chloride (PVC) tube (6 cm wide by 18 cm long) that had been perforated with three horizontal slits for termite access (Figure 1); each tube was capped at ground level. We installed these IGMs in two concentric rings encircling each structure, with the inner ring positioned ~1 m from the foundation and the two rings ~3 m apart. (Minor deviations to this layout occurred in Texas and Ohio when property boundaries and/or construction work precluded the entire second ring.) Stations were spaced ~2 m apart in the inner ring and ~3 m apart in the outer ring. We installed an average of 39.5 IGMs on each property (41 in Texas, 36.5 in North Carolina, and 41 in Ohio). We subsequently inspected all IGMs (and collected termite samples) on approximately a monthly basis, with an infestation level of ≥10% as the threshold for treatment.

Altriset^®^ Termiticide (Syngenta Crop Protection LLC, Greensboro, NC, USA), which is formulated as a water-based suspension concentrate, was applied as a 0.05% finished solution following the manufacturer’s label instructions for post-construction termite treatment [20]. All treatments were carried out by a licensed pest management professional (PMP). A defined treatment (exterior perimeter with limited interior treatment at infested sites, including foam applications as necessary) was conducted at all houses except two in North Carolina. NC-2 and NC-4 were treated using a conventional complete treatment wherein the termiticide is applied to the interior and exterior perimeter to form a continuous chemical barrier. The label [20] describes defined and complete treatment procedures for various foundation types. Note that IGMs were never inside the treated zone.

Following the chlorantraniliprole treatment, we inspected each structure and all IGMs at ~1–3 months after treatment (MAT) and then approximately quarterly for 2 years. A sample was collected for genotyping each time that termites were found in the structure or IGMs.

We genotyped 10 workers (or all if fewer were present) from each termite sample to determine their colony affiliation. Using established methods [21,22], we extracted the DNA of individual workers for microsatellite genotyping at two loci, *Rf 24-2* and *Rf 21-1*. We used Genepop on the Web, a population genetics software package originally developed by Raymond and Rousset [23], to compare genotypes from all pairs of termite samples. We considered groups of workers to belong to the same colony if they all shared the same genotypes and their genotype frequencies did not significantly differ (*p* > 0.05 with a Bonferroni correction) based on an exact test of genotypic differentiation. This method previously has been used to determine colony affiliation of *R. flavipes* following an insecticide treatment [22,24].

At each property, the infesting colony consisted of termites with the same genotype/genotype frequency that were detected in/on the structure and any surrounding IGMs. Landscape colonies consisted of genotypically similar termites detected in IGMS but never in/on the structure.

The foraging area of each colony was estimated using SketchAndCalc™ Area Calculator version 1.1.0 (Icalc, Inc., Palm Coast, FL, USA). We prepared a scale diagram of each structure and its associated IGMs then drew a polygon that encompassed all known termite-infested sites (colony specific) in the structure and/or landscape. The polygon was created using the shortest straight line distance between infestation points, assuming a buffer radius of ~1 m per point. For landscape colonies, none of which were found in or on the structure, lines that encompassed infested IGMs were positioned outside of the structure boundaries. Solitary infested IGMs were estimated to encompass an area of 2 m^2^ and lines were drawn parallel to the structure.

## 3. Results

Microsatellite markers indicated that each of the structures was infested by only one *R. flavipes* colony. In the three states, the single infesting colony was collected during pre-treatment inspections at one or more sites in or on the foundation as well as in numerous IGMs in the landscape. Chlorantraniliprole provided effective structural protection as termite activity in and on the majority of structures ceased within less than ~1 month post-treatment, and no termites were detected in the structures for the 2-year study duration.

### 3.1. College Station, TX Properties (Monolithic Slab Foundation)

**Figure 2:**
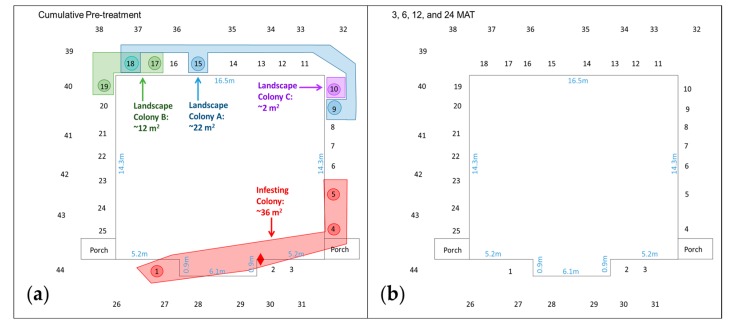
Estimated foraging area of the *R. flavipes* infesting colony (red) and Landscape Colonies A (blue), B (green), and C (purple) before and after a chlorantraniliprole treatment at TX-3 in College Station, TX. Sequential numbers represent in-ground monitors in the landscape. Diamond represents termites collected from a shelter tube on the monolithic slab foundation. Observed termite activity: (**a**) During the cumulative pre-treatment period; (**b**) At 3, 6, 12, and 24 months after treatment (MAT).

For the duration of the post-treatment period (1–24 MAT), members of the infesting colony at TX-3 (Figure 2) and TX-4 were not found in the structure or any IGMs (Table 1). The apparent absence of both infesting colonies contrasts with their pre-treatment foraging area, which was estimated at 36 and 192 m^2^, respectively.

The TX-1 Infesting Colony likewise was no longer found in the structure during any post-treatment inspections (1–19 MAT), but it continued to be periodically found in IGMs in the landscape (Table 1). However, its observed footprint was considerably reduced compared to the 2-month pre-treatment period when TX-1 colony members were found in the structure and eight different IGMs, encompassing a foraging area of approximately 138 m^2^ (Table 1).

The estimated foraging area of the TX-2 Infesting Colony’s was 36 m^2^ prior to treatment, and afterward, termites were never detected in any IGMs (Table 1). However, members of the infesting colony persisted in the structure as a few alates and workers were found near a bedroom window at 5 MAT even though there were no signs of water leaks, and moisture meter readings (10%–15%) were not indicative of termite activity [25]. After a supplemental spot-treatment using 41 L of chlorantraniliprole, no further termite activity was observed in the structure (Table 1).

Colonies exclusively associated with the landscape (no colony members detected attacking the structure) were evident at TX-1 and TX-3 during the pre- and/or post-treatment periods (Table 1). TX-1 Landscape Colony A was found at IGMs #1 and #27 (estimated foraging area of 13 m^2^) at 1 month prior to treatment and it subsequently was found at IGM #1 during four post-treatment inspections. Additionally, three new landscape colonies (B, C, and D) were observed, each in a single monitor, during the 19-month post-treatment inspections of TX-1. Of the four landscape colonies observed at TX-1, only Landscape Colony A was found at the final inspection (Table 1). At TX-1, different colonies occupied IGMs #1 and #32 at different inspection times. For example, IGM #1 was occupied multiple times by Landscape Colony A during the pre- and post-treatment periods, but it was occupied by the infesting colony at 17 MAT. Similarly, IGM #32 was occupied by the infesting colony during the pre- and post-treatment periods, and it was occupied by Landscape Colony C at 9 MAT (Table 1).

At TX-3, three landscape colonies (A, B, and C) were found during the 1.5-month pre-treatment period (Figure 2a). Landscape Colonies A and B were found in three IGMs, providing an estimated foraging area of 22 and 12 m^2^, respectively (Table 1). Landscape Colony C was found in a single IGM, #10. Landscape Colonies A and B occupied IGM #18 at various times, but apparently not simultaneously based on microsatellite data. The three landscape colonies at TX-3 were no longer detected after the chlorantraniliprole treatment (Figure 2b).

### 3.2. Raleigh, NC Properties (Crawl Space Foundation)

**Figure 3:**
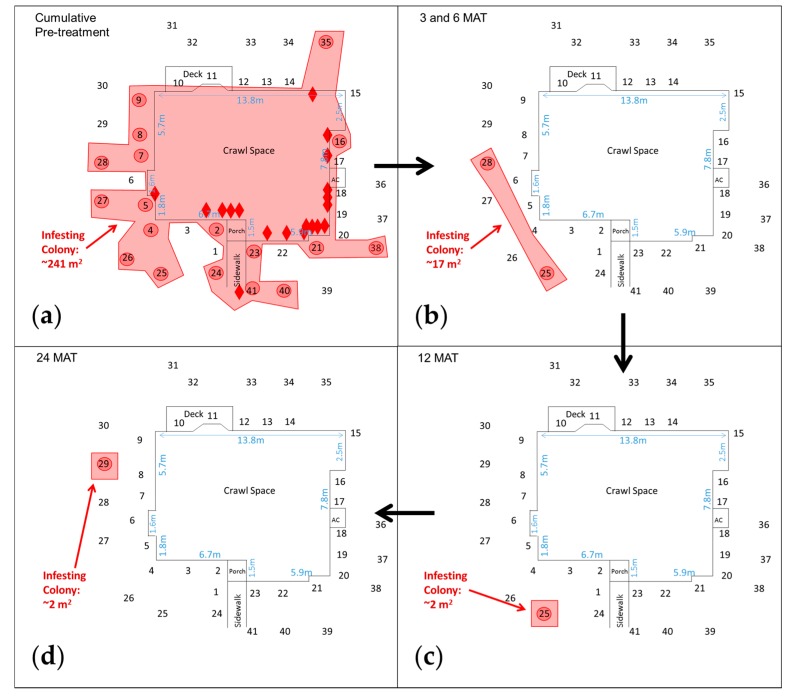
Estimated foraging area of the *R. flavipes* infesting colony before and after a chlorantraniliprole treatment at NC-2 near Raleigh, NC. Sequential numbers represent in-ground monitors in the landscape. Diamonds represent termites collected on the crawl space foundation, from shelter tubes, and in auxiliary wooden stakes. Observed termite activity: (**a**) During cumulative pre-treatment period; (**b**) At 3 and 6 months after treatment (MAT); (**c**) At 12 MAT; (**d**) At 24 MAT.

At the 1 month post-treatment inspection and thereafter, termites from the infesting colony were no longer evident in the structure at NC-2 (Figure 3) and NC-4 (Table 2). A similar scenario was observed at NC-3, but with a 1-month lag, as members of the infesting colony were last observed in the structure at 2 MAT. The infesting colony’s post-treatment fate in the surrounding landscape showed variability among these properties, ranging from being apparently reduced (NC-2 and NC-3) to apparently eliminated (NC-4) during the post-treatment period. At NC-2 and NC-3, the observed footprint of each infesting colony was reduced to a small area in the landscape after the chlorantraniliprole treatment. For example, the NC-2 Infesting Colony occupied eighteen sites in the structure and eighteen IGMs during an 8-month pre-treatment period (Figure 3a) compared to just one or two IGMs at several post-treatment inspections (Figure 3b,c,d); its estimated foraging area was reduced from 241 m^2^ pre-treatment to 2 m^2^ at 24 MAT (Table 2). At NC-3, the infesting colony was found in a shelter tube at 1 MAT and in a tree stump near the structure at 18 MAT when the study was prematurely concluded due to a persistent indoor water leak (Table 2). Members of the NC-4 Infesting Colony were last found in IGMs in the landscape at 6 MAT.

The NC-1 Infesting Colony was observed at four sites in the structure and in two IGMs prior to treatment and it was not found in any IGMs afterwards (Table 2). Termites from the infesting colony persisted in the structure following the treatment (Table 2), but this structure had moisture problems. In addition to an apparent water leak from a bathroom shower, the crawl space was very humid. A vapor barrier and additional vents were installed at 5 MAT, but wood moisture meter readings were high, ranging from 18–24%, at 6 MAT. Termites constructed new shelter tubes attempting to tunnel downward to reconnect with the soil. Within 2 months of a supplemental spot-treatment using 7.6 L of chlorantraniliprole, no further evidence of the infesting colony was observed in the NC-1 structure, with colony members last found in a single joist at 8 MAT (Table 2).

Colonies exclusively associated with the landscape were evident at NC-1, NC-3, and NC-4 (Table 2). At both NC-1 and NC-4, a single landscape colony was observed in only one IGM during pre-treatment inspections. NC-1 Landscape Colony A was seen twice, about midway through the pre-treatment period (November 2012) but not subsequently. NC-4 Landscape Colony A showed up in IGM #27 during early August 2013 prior to treatment and it subsequently expanded its range to occupy four additional IGMs during the 2-year post-treatment period. Furthermore, a new NC-4 landscape colony, B, was observed in IGM #30 at every post-treatment inspection. At NC-3, two landscape colonies, A and B, were each found once during the 10-month pre-treatment period and five landscape colonies (A, B, C, D, and E) were sporadically detected after treatment: Landscape Colony A was last found at 12 MAT; Landscape Colonies C and E were each found in a single IGM only at 1 MAT and 18 MAT, respectively; whereas Landscape Colonies B and D were found for the duration of the 18-month post-treatment period.

### 3.3. Columbus, OH Properties (Basement Foundation)

**Figure 4:**
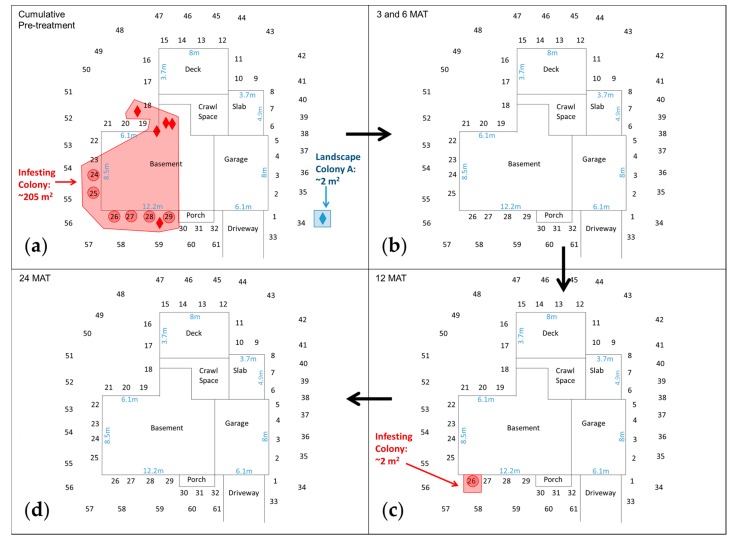
Estimated foraging area of the *R. flavipes* infesting colony (red) and Landscape Colony A (blue) before and after a chlorantraniliprole treatment at OH-1 in Columbus, OH. Sequential numbers represent in-ground monitors in the landscape. Diamonds represent termites collected in or on the basement foundation or in wood in the landscape. Observed termite activity: (**a**) During cumulative pre-treatment period; (**b**) At 3 and 6 months after treatment (MAT); (**c**) At 12 MAT; (**d**) At 24 MAT.

Pre- and post-treatment data for Ohio indicated that OH-1, OH-2, and OH-3 showed no further evidence of termite activity in/on the structures within 1 month after a chlorantraniliprole treatment (Table 3). OH-4 was the only structure with any surviving termites indoors, and the majority was dead and dying at 1 MAT and no live termites were observed thereafter. Members of the OH-1 Infesting Colony (Figure 4a) were last found in IGMs at 12 MAT (Figure 4c,d).

Despite no longer attacking the structure at OH-2, OH-3, and OH-4, each of the infesting colonies persisted in the landscape in some IGMs during the 2-year post-treatment period (Table 3). However, the infesting colony’s observed footprint at OH-2 and OH-4 was reduced to a small area in the landscape at the conclusion of our study. For example, the OH-2 Infesting Colony was found in three IGMs at the final inspection during August 2015 as compared to its occupancy of six IGMS and the structure during a 2.5-month pre-treatment period 2 years earlier. Hence, its estimated foraging area was reduced from 132 m^2^ pre-treatment to 31 m^2^ at 25 MAT. The OH-4 Infesting Colony had occupied the structure and nine different IGMs during a 3-month pre-treatment period, but it was found in only three IGMs at 1 MAT and then not again until 15 to 24 MAT when it was sporadically found in one or two IGMs. The estimated foraging area of the OH-4 Infesting Colony was reduced from 110 m^2^ pre-treatment to 24 m^2^ at 24 MAT. OH-3 was the only property where termites occupied comparable numbers of IGMs during the pre- and post-treatment periods. Nonetheless, the OH-3 infesting colony’s estimated foraging area was reduced from 118 m^2^ pre-treatment to 32 m^2^ at 24 MAT (Table 3).

Microsatellite data revealed one colony exclusively associated with the landscape at both OH-1 (Figure 4) and OH-4 (Table 3). These landscape colonies had a much smaller observed footprint than the corresponding infesting colony; they were found in or near a single IGM during one pre-treatment inspection. Each was found once after the treatment and near its original location.

## 4. Discussion

Our results indicated that a single chlorantraniliprole termiticide treatment had colony-level effects and provided effective structural protection irrespective of foundation type (monolithic slab, crawl space, or basement) in three states. At the majority of properties (83.3%), subterranean termite activity was no longer detected in the structure within 1 or 2 MAT and thereafter for the 2-year study duration (Table 1, Table 2 and Table 3). The supplemental spot-treatment at TX-2 and NC-1 also corresponded with the apparent elimination of the infesting colony from each structure and IGMs in the landscape.

After treatment, the infesting colony appeared to be eliminated from both the structure and IGMs in the landscape at some properties. For example, there was no further evidence of the TX-3 and TX-4 infesting colony at 1 MAT and thereafter (Table 1). At NC-4 and OH-1, members of the infesting colony were last detected in IGMs at 6 months (Table 2) and 12 months (Table 3), respectively, and these infesting colonies also were presumably eliminated. During the post-treatment period, the infesting colony appeared to be eliminated from the structure and IGMs at 50% of the overall properties (6 of 12). However, it is possible that some infesting colonies were not detected by our sampling methods, moved out of the study site, or died of other causes. As discussed below, if the infesting colonies were not eliminated, the most plausible alternative scenario is that colony size was so greatly reduced that termites were no longer detected by our IGMs in these residential landscapes.

When the infesting colony persisted at IGMs following treatment (TX-1, NC-2, NC-3, OH-2, OH-3, and OH-4), its estimated foraging area typically was reduced to a small area in the landscape. The estimated pre-treatment foraging area of these six colonies averaged (±SD) 126 ± 58 m^2^, and the average area at last observation was 16 ± 15 m^2^, which is a decrease of approximately 88 ± 38%. For example, the TX-1 Infesting Colony’s pre-treatment foraging area was 138 m^2^, but after treatment, colony members were detected in only a few IGMs at intermittent inspections and in none at the final inspection (Table 1). Overall, the infesting colony appeared to be reduced in the landscape at 42% of 12 properties, and OH-3 was the only property where the infesting colony continued to occupy similar numbers of IGMs during the pre- and post-treatment periods. Nonetheless, at OH-3, the infesting colony’s estimated foraging area was reduced from 118 m^2^ pre-treatment to 32 m^2^ at 24 MAT (Table 3). Given our intensive sampling timetable, we did find further evidence of some infesting colonies in the landscape after rather large gaps in time, i.e., TX-1, NC-3, OH-2, and OH-4.

Other nonrepellent soil termiticides similarly have caused decline or apparent elimination of infesting colonies of *Reticulitermes* spp. For example, imidacloprid caused some infesting colonies to disappear and others to reduce their foraging area to such an extent that only colony remnants were periodically detected in Raleigh, NC residential landscapes [26]. Treatment with fipronil was characterized by apparent elimination of all infesting colonies, without remaining remnants, also in Raleigh, NC residential landscapes [22].

In our study, a supplemental spot-treatment was conducted only at one house in Texas (TX-2) at 5 MAT and at one house in North Carolina (NC-1) at 6 MAT. In the NC-1 crawl space, high levels of wood moisture, high humidity, and a water leak likely had created favorable aboveground conditions wherein termites did not need to forage through the chlorantraniliprole treated soil. Note that the label cautions: “Altriset may not be completely effective unless conducive conditions (i.e., moisture problems, direct wood to soil contact) are corrected”. In contrast, at TX-2, moisture levels were normal in the vicinity of the few workers and alates observed at 5 MAT and there was no obvious explanation for the reduced but ongoing termite activity. Nonetheless, the supplemental treatment resulted in the apparent elimination of the infesting colony from both properties. 

Landscape colonies (non-infesting) were detected in only 1–2 IGMs at many properties, either pre- or post-treatment, yet their overall foraging area obviously included additional sites that we did not sample. Even when landscape colonies occupied numerous IGMs, it is difficult to ascertain whether the treatment had area-wide effects. In residential landscapes in Raleigh, NC, where sampling was done in monitors that were >6.5 m from the structure and in surrounding natural areas, 71% of untreated colonies were re-detected over a period of 9.5 ± 2.7 months (range, 5.3 to 14.4) prior to an imidacloprid treatment [26].

The number of colonies found exclusively in the landscape (not attacking the structure) varied among the states, with the largest number of colonies in Texas (0–4) and North Carolina (0–5) as compared to 0–1 in Ohio, the most northern state. Note that we likely underestimated the total number of colonies at each property since we surveyed for termites only close to each structure. In North Carolina, average sized residential properties (measuring 1854 m^2^) infested with termites were estimated to have a mean of 9.4 *R. flavipes* colonies [21]. In Massachusetts, an undisturbed site had approximately seven colonies per ha (three colonies per acre) [27], thereby leading Parman and Vargo [21] to suggest that *R. flavipes* may have much lower colony densities in the northern part of its range as compared to North Carolina. Our data support their hypothesis. Our finding that each structure was infested by a single *R. flavipes* colony also is consistent with other studies where this is the norm [21,22,24,26].

## 5. Conclusions

In conclusion, through genetic identification of individual termite colonies and intensive monitoring of infested structures and the surrounding landscape, our evidence suggests that chlorantraniliprole effectively protected structures in three states. Our results indicated that this soil termiticide severely impacted or possibly eliminated *R. flavipes* infesting colonies, thereby demonstrating its colony-level effects.

## Figures and Tables

**Table 1:** Sites occupied by the infesting colony and the estimated foraging area prior to and after a chlorantraniliprole treatment at four monolithic slab structures in College Station, TX. Data also are provided for landscape colonies that were found solely in in-ground monitors (IGMs) at two of these properties. Colony designations (Infesting Colony, A, B, C, etc.) are unique to each property.

Property {Area of Structure (sq m)}, Study Dates ^1^	IGMs ^2^	Cumulative Pre-Treatment Termite Activity ^3^ {Estimated Foraging Area (sq m)}	Treatment Date, Volume (L)	Post-Treatment Termite Activity ^4^ {Estimated Foraging Area (sq m) at Final Inspection}
TX-1 ^5^ {109}, 6-VIII-12 – 7-V-14	#1–#27 + #28–#40	Infesting Colony {138} 1 tube on foundation; IGMs #14, #22–#25, #27, #32, #35 Landscape Colonies in IGMs Colony A {13}: #1, #27	15-X-12, 307	Infesting Colony {0} No evidence in structure; IGMs #1_17MAT_, #32_17,18MAT_, #39_9MAT_ Landscape Colonies in IGMs Colony A {2}: #1_7,8,18,19MAT_; Colony B {0}: #31_7,8MAT_; Colony C {0}: #32_9MAT_; Colony D {2}: #20_17MAT_
TX-2 {146}, 6-VIII-12 – 4-XII-14	#1–#27 + #28–#36	Infesting Colony {36} 2 tubes on foundation; IGMs #1, #4, #6	9-XI-12, 265 ^6^	Infesting Colony {0} In window ^7^ @ 5 MAT; None in IGMs
TX-3 {278}, 10-VIII-12 – 4-XII-14	#1–#25 + #26–#44	Infesting Colony {36} 1 tube on foundation; IGMs #1, #4, #5 Landscape Colonies in IGMs Colony A {22}: #9, #15, #18; Colony B {12}: #17–#19; Colony C {2}: #10	21-IX-12, 337	Infesting Colony {0} No evidence in structure; None in IGMs Landscape Colonies in IGMs {0} None detected
TX-4 {278}, 3-XII-12 – 30-VII-15	#1–#26 + #27–#44	Infesting Colony {192} 1 tube on foundation; IGMs #1–#4, #14	12-XI-13, 326	Infesting Colony {0} No evidence in structure; None in IGMs

^1^ From study inception through conclusion, typically at ~24 months after treatment (MAT); ^2^ IGMs installed in two concentric rings surrounding the structure, with # indicating the sequential number of an individual monitor. IGMs in the inner + outer rings are on separate lines, respectively; ^3^ Structure and IGMs inspected approximately monthly; ^4^ Structure and IGMs inspected at 1–3 MAT and then approximately quarterly for 2 years; ^5^ Study was concluded at 19 MAT rather than 24 MAT because pest management professional erroneously spot-treated the bathroom with different termiticide as a precautionary measure due to a leaking toilet; ^6^ Spot-treatment near the bedroom window with 37 L applied to soil along the exterior perimeter and 4 L foam applied inside the wall void on 1 May 2013; ^7^ ~5 to 10 live *R. flavipes* workers and swarmers on 29 April 2013, but none seen during spot-treatment.

**Table 2:** Sites occupied by termites from the infesting colony prior to and after a chlorantraniliprole treatment at four crawl space structures near Raleigh, NC. Data also are provided for colonies that were found solely in the landscape at three of these properties. Colony designations (Infesting Colony, A, B, C, etc.) are unique to each property.

Property {Area of Structure (sq m)}, Study Dates ^1^	IGMs ^2^	Cumulative Pre-Treatment Termite Activity ^3^ {Estimated Foraging Area (sq m)}	Treatment Date, Volume (L)	Post-Treatment Termite Activity ^4^ {Estimated Foraging Area (sq m) at Final Inspection}
NC-1 {190}, 8-VIII-12 – 17-IV-15	#1–#21 + #22–#39	Infesting Colony {44} Shelter tubes in crawl space (1, 2, 3, 4); IGMs #5, #7 Landscape Colonies in IGMs Colony A {2}: #4	26-IV-13, 587 ^5,6^	Infesting Colony {0} Tubes 1_3MAT_, 2_2MAT_, 3_4,6MAT_, 4_2–4MAT_, 5_3MAT_, 6_3MAT_, 7_3–6MAT_, 8_5MAT_; Joists 1_2MAT_, 2–3_4MAT_, 4_8MAT_ Landscape Colonies in IGMs {0} None detected
NC-2 {129}, 7-VIII-12 – 22-IV-15	#1–#23 + #24–#41	Infesting Colony {241} 4 auxiliary wooden stakes; 14 tubes on interior and exterior of foundation; IGMs #2, #4, #5, #7–#9, #16, #21, #23–#28, #35, #38, #40, #41	18-IV-13, 776 ^7^	Infesting Colony {2} No evidence in structure; IGMs #25_1–3,6,9,12MAT_, #26_1MAT_, #28_1–3,6,15,18MAT_, #29_18,21,24MAT_, #35_1MAT_, #40_1MAT_
NC-3 ^8^ {136}, 12-VI-12 – 17-X-14	#1–#16 + #17–#35	Infesting Colony {40} Tubes in crawl space (2, 3, 4); 2 floor joists in crawl space; IGM #22 Landscape Colonies in IGMS Colony A {4}: #22; tree stump Colony B {2}: #23	19-IV-13, 598 ^5^	Infesting Colony {2} Tube 2_1MAT_; tree stump_18MAT_ Landscape Colonies in IGMs Colony A {0}: #22_1,2,6,9,12MAT_; Colony B {2}: #23_1-18MAT_; Colony C {0}: #20_1MAT_; Colony D {2}: #27_2,3MAT_, #25_2,3,6,12,15,18MAT_, #26_6,9MAT_ Colony E {2}: #28_18MAT_
NC-4 {106}, 13-VI-13 – 8-VIII-15	#1–#14 + #15–#31	Infesting Colony {74} 3 tubes in crawl space; IGMs #2, #9, #13, #22, #25, #26 Landscape Colonies in IGMs Colony A {2}: #27	27-VIII-13, 731 ^7^	Infesting Colony {0} No evidence in structure; IGMs #25_1-3MAT_, #26_1–3,6MAT_ Landscape Colonies in IGMs Colony A {2}: #4_1MAT_, #11_2,3,MAT_, #12_12MAT_, #27_1–3,9,12,15,24MAT_, #28_1–3,6,12,21MAT_; Colony B {2}: #30_1–24MAT_

^1–4^ See Table 1; ^5^ Defined treatment; ^6^ Altriset^®^ Foam spot-treatment (7.6 L) of damp floor joists on 18 October 2013; ^7^ Complete treatment; ^8^ Study was concluded at 18 MAT rather than 24 MAT because of a persistent water leak in crawl space that the homeowner did not remedy.

**Table 3:** Sites occupied by termites from the infesting colony prior to and after a chlorantraniliprole treatment at four basement structures in Columbus, OH. Data also are provided for landscape colonies that were found solely in or near in-ground monitors (IGMs) at two of these properties. Colony designations (Infesting Colony, A, B, C, etc.) are unique to each property.

Property {Area of Structure (sq m)}, Study Dates ^1^	IGMs ^2^	Cumulative Pre-Treatment Termite Activity ^3^ {Estimated Foraging Area (sq m)}	Treatment Date, Volume (L)	Post-Treatment Termite Activity ^4^ {Estimated Foraging Area (sq m) at Final Inspection}
OH-1 {185}, 25-VI-12 – 12-XII-14	#1–#32 + #33–#61	Infesting Colony {205} Wood debris near house; 2 tubes; 3 auxiliary monitors in basement; IGMs #24‒#29 Landscape Colonies in IGMs Colony A {2}: Near #34	20-IX-12, 1079	Infesting Colony {0} No evidence in structure; IGMs #18_1MAT_, #25_1MAT_, #26_1,2,7,9,12MAT_ Landscape Colonies in IGMs Colony A {0}: #1_7MAT_
OH-2 {119}, 13-V-13 – 12-VIII-15	#1–#20 + #21–#40	Infesting Colony {132} Kitchen wall; 6 tubes on foundation; IGMs #6, #11, #28, #31, #35, #37	25-VII-13, 757	Infesting Colony {31} No evidence in structure; IGMs #7_2,12MAT_, #13_25MAT_, #16–#17_12MAT_, #29_12MAT_, #30_25MAT_, #31_17MAT_, #35–36_2MAT_, #37_17,21,25MAT_
OH-3 {136}, 26-VII-13 – 15-IX-15	#1–#17 + #18–#27	Infesting Colony {118} Alates in upstairs bathroom; 2 auxiliary monitors in basement; tubes on foundation; tree stump near structure; IGMs #1, #3, #5, #10, #11, #21–#23	13-IX-13, 908	Infesting Colony {32} No evidence in structure; IGMs #3_1,2,3,8,19,21,24MAT_, #4_8,10,15,21,24MAT_, #6_2,3MAT_, #5_1,2,3,8,10MAT_, #7_10,15,24MAT_, #8_1,2,3,10,19,21MAT_, #9_1,2,21MAT_, #10_8MAT_, #11_1,8,10,15,21,24MAT_, #12_10MAT_, #18_21MAT_, #21_1MAT,_ #22_19,21,24MAT_, #23_1MAT_
OH-4 {91}, 13-VIII-13 – 15-IX-15	#1–#21 + #22–#36	Infesting Colony {110} Auxiliary monitors in basement (1, 2, 4, 5); tubes on foundation; IGMs #8, #12, #16–#19, #30, #31, #33 Landscape Colonies in IGMs Colony A {2}: #7	13-IX-13, 757	Infesting Colony {24} Auxiliary monitors ^5^ 4–5_1MAT_; IGMs #12_1MAT_, #17_21,24MAT_, #30_1MAT_, #31_18,21MAT_, #32_24MAT_, #33_1,15MAT_ Landscape Colonies in IGMs Colony A {0}: #7_1MAT_

^1–4^ See Table 1; ^5^ Majority of termites dead or dying.
